# Supplementary material for: Iterative improvement in the automatic modular design of robot swarms
Source: PeerJ Comput Sci. 2020 Dec 7;6:e322. doi: 10.7717/peerj-cs.322 (PMC7924708; doi:10.7717/peerj-cs.322)
Supplement: Supplemental Information 3 [file peerj-cs-06-322-s003.zip › argos3/doc/api/standalone/a00430_source.html]

ARGoS: plugins/robots/eye-bot/simulator/pointmass3d\_eyebot\_model.cpp Source File


- Main Page
- Related Pages
- Namespaces
- Classes
- Files

- File List
- File Members

# plugins/robots/eye-bot/simulator/pointmass3d\_eyebot\_model.cpp

Go to the documentation of this file.

```
00001 
00007 #include "pointmass3d_eyebot_model.h"
00008 #include <argos3/core/utility/logging/argos_log.h>
00009 #include <argos3/core/utility/math/cylinder.h>
00010 #include <argos3/core/simulator/simulator.h>
00011 #include <argos3/core/simulator/space/space.h>
00012 #include <argos3/plugins/simulator/physics_engines/pointmass3d/pointmass3d_engine.h>
00013 
00014 namespace argos {
00015 
00016    static const Real     BODY_HEIGHT  = 0.566f;
00017    static const Real     BODY_RADIUS  = 0.25f;
00018    static const Real     BODY_MASS    = 1.00f;
00019    static const Real     BODY_INERTIA = 0.01f;
00020    static const CVector3 POS_K_P(20.0f, 20.0f, 20.0f);
00021    static const CVector3 POS_K_D(10.0f, 10.0f, 10.0f);
00022    static const Real     YAW_K_P = 0.5f;
00023    static const Real     YAW_K_D = 0.1f;
00024    static const CVector3 VEL_K_P(20.0f, 20.0f, 20.0f);
00025    static const CVector3 VEL_K_D(10.0f, 10.0f, 10.0f);
00026    static const Real     ROT_K_P = 0.5f;
00027    static const Real     ROT_K_D = 0.1f;
00028 
00029    /****************************************/
00030    /****************************************/
00031 
00032    CPointMass3DEyeBotModel::CPointMass3DEyeBotModel(CPointMass3DEngine& c_engine,
00033                                                     CEyeBotEntity& c_eyebot) :
00034       CPointMass3DQuadRotorModel(c_engine,
00035                                  c_eyebot.GetEmbodiedEntity(),
00036                                  c_eyebot.GetQuadRotorEntity(),
00037                                  BODY_HEIGHT,
00038                                  BODY_RADIUS,
00039                                  BODY_MASS,
00040                                  BODY_INERTIA,
00041                                  POS_K_P,
00042                                  POS_K_D,
00043                                  YAW_K_P,
00044                                  YAW_K_D,
00045                                  VEL_K_P,
00046                                  VEL_K_D,
00047                                  ROT_K_P,
00048                                  ROT_K_D) {
00049       RegisterAnchorMethod<CPointMass3DEyeBotModel>(
00050          GetEmbodiedEntity().GetAnchor("perspective_camera"),
00051          &CPointMass3DEyeBotModel::UpdatePerspectiveCameraAnchor);
00052    }
00053 
00054    /****************************************/
00055    /****************************************/
00056 
00057    void CPointMass3DEyeBotModel::UpdatePerspectiveCameraAnchor(SAnchor& s_anchor) {
00058       s_anchor.Position = m_cPosition;
00059    }
00060 
00061    /****************************************/
00062    /****************************************/
00063 
00064    REGISTER_STANDARD_POINTMASS3D_OPERATIONS_ON_ENTITY(CEyeBotEntity, CPointMass3DEyeBotModel);
00065 
00066    /****************************************/
00067    /****************************************/
00068 
00069 }
```

---

Generated on 10 Jul 2018 for ARGoS by 
 1.6.1
